# Outdoor physical activity in traditional and newly designed preschools: a cross-sectional study

**DOI:** 10.1186/s12889-025-23455-z

**Published:** 2025-06-26

**Authors:** Andreas Fröberg, Therese Eskilsson, Annika Manni, Jonas Markström

**Affiliations:** 1 Department of Food and Nutrition and Sport Science, University of Gothenburg, Läroverksgatan 5, PO Box 300, Gothenburg, SE-405 30 Sweden; 2 Department of Community Medicine and Rehabilitation, Umeå University, Umeå, Sweden; 3 Department of Applied Educational Sciences, Umeå University, Umeå, Sweden

**Keywords:** Environmental, Outdoor education, Physical activity, Preschool, Play environments

## Abstract

**Background:**

Outdoor time and play are crucial for children’s learning and development, impacting various physical, cognitive, and social aspects of their well-being and socio-emotional growth. In Sweden, new preschools are increasingly built as multi-story facilities, accommodating larger cohorts of children while often reducing outdoor space. This trend raises concerns about the quality of outdoor environments and their impact on physical activity (PA) opportunities. This study aims to explore outdoor total PA and PA levels among children in traditionally and newly designed preschools across varied outdoor play environments. The primary research question was: How does total outdoor PA differ among children attending traditionally designed versus newly designed preschools, and across different types of outdoor play environments? The hypothesis was that children’s total outdoor PA is higher in preschools with higher-quality outdoor play environments, and, among preschools of similar quality, those with traditional designs are associated with higher PA levels than newly designed ones. The secondary research question was: How do outdoor PA levels differ among children attending traditionally designed versus newly designed preschools? The hypothesis was that children attending traditionally designed preschools engage in higher levels of outdoor PA than those attending newly designed preschools.

**Methods:**

This cross-sectional study involved 106 children aged 3-5-year-olds from six (3 traditional and 3 newly designed) strategically selected preschools in Sweden. Data for total PA was collected with accelerometers (ActiGraph GT3X+) among 106 children. Data for PA level (*n* = 371 observations) was collected through observations. The preschool outdoor play environments were assessed using the Outdoor Play Environment Categories (OPEC). Multiple regression was used to assess how preschool type (traditional/newly designed) and OPEC predicted total PA. Chi-square test was used to explore differences in PA levels.

**Results:**

The result showed that children in newly designed preschools (*p* = 0.019 for forenoon and *p* = 0.049 for afternoon) and children in preschools with higher OPEC (*p* < 0.001 for forenoon and *p* < 0.001 for afternoon) had higher outdoor total PA than their children peers. In contrast, observations showed that PA levels differed among children in traditional and newly designed preschools (*p* = 0.005), with those at traditional preschools having higher proportions of moderate movements.

**Conclusions:**

The present study showed that children in newly designed preschools, and children in preschools with higher-quality outdoor play environment, had higher outdoor total PA than their children peers. In addition, that children in traditional preschools had higher proportions of moderate movements compared to newly designed preschools. These results emphasise the importance of balancing environmental design and unstructured PAs to support diverse and engaging outdoor PA opportunities for children. Further studies should explore how social dynamics, spatial organization, and activity flow within newly designed preschools across different outdoor play environments contribute to higher total PA and different PA levels among children.

## Background

Outdoor time and play are crucial for children’s learning and development, impacting various physical, cognitive, and social aspects of their well-being and socio-emotional growth [1–4]. However, contemporary changes in preschool design, driven by urbanization and population growth, may raise concerns about the quality and accessibility of outdoor play environments for preschool children’s physical activity (PA). Research consistently highlights the benefits of outdoor play environment and its positive association with PA among children, especially in traditional preschool settings with spacious, well-equipped outdoor areas [1, 3, 4, 5, 6]. For example, larger playgrounds, low child density, and the availability of portable play equipment have been associated with higher levels of PA in preschool settings (5–6). Nonetheless, previous research suggests a dynamic interaction between outdoor environments and children’s play, highlighting the complex nature of playground design. Children may utilize diverse spaces and materials during outdoor play, with activities such as biking, climbing, running and tumbling constituting a large proportion of playtime [7]. Despite these insights, there is a significant gap in understanding how newly designed preschools– often constrained by limited space and construction norms– affect outdoor PA and overall opportunities for outdoor play among children.

In Sweden, where preschool attendance is exceptionally high, outdoor play is not only a widely embraced practice but also a key component of the national curriculum (8–9). Approximately 85–95% of children aged one to five years in Sweden are enrolled in preschool, with outdoor time forming a substantial part of the daily schedule [8, 10]. Evidence suggests that children in Swedish preschools are more physically active during outdoor time compared to indoor, aligning with findings from international research (11–12). To support outdoor play, the Swedish National Board of Housing, Building and Planning [13] recommends that preschools provide accessible outdoor areas of at least 3000 m², or approximately 40 m² per child. However, these recommendations may not always be feasible in newly designed preschools, particularly in densely populated urban areas.

Recent trends in preschool design in Sweden reflect the challenges posed by urbanization and space limitations. Many new preschools are built as multi-story facilities and often accommodate significantly larger cohorts of children while maintaining the same or even reduced outdoor space. These designs may have implications for the quality of outdoor environments, as larger groups of children sharing the same or smaller outdoor areas may limit opportunities for PA. This may include not only total PA, but also PA levels (the behavioural form of PAs at different levels, such as stationary, slow-easy, moderate, and fast) and type (the typography of PAs, such as jumping/skipping, running, swinging etc.). Some early qualitative findings suggest that newly designed preschools often lack diverse outdoor equipment and natural elements, such as trees, uneven terrain, and unstructured play spaces, which are known to encourage varied and creative PA [14].

Despite some early qualitative findings [14], quantitative research comparing outdoor PA between traditional and newly designed preschools with varied outdoor play environments remains limited. This lack of evidence presents a challenge for policymakers and educators seeking to optimize preschool designs to support PA and developmental needs among children. Using a quantitative approach, this study aims to explore outdoor total PA and PA levels among children in traditionally and newly designed preschools across varied outdoor play environments. The primary research question (RQ-1) that guided the study was:

- How does total outdoor PA differ among children attending traditionally designed versus newly designed preschools, and across different types of outdoor play environments?
- Hypothesis: Children’s total outdoor PA is higher in preschools with higher-quality outdoor play environments, and, among preschools of similar quality, those with traditional designs are associated with higher PA levels than newly designed ones.

The secondary research question (RQ-2) was:

- How do outdoor PA levels differ among children attending traditionally designed versus newly designed preschools?
- Hypothesis Children attending traditionally designed preschools engage in higher levels of outdoor PA than those attending newly designed preschools.

## Materials and methods

### Study design and population

**Fig. 1:**
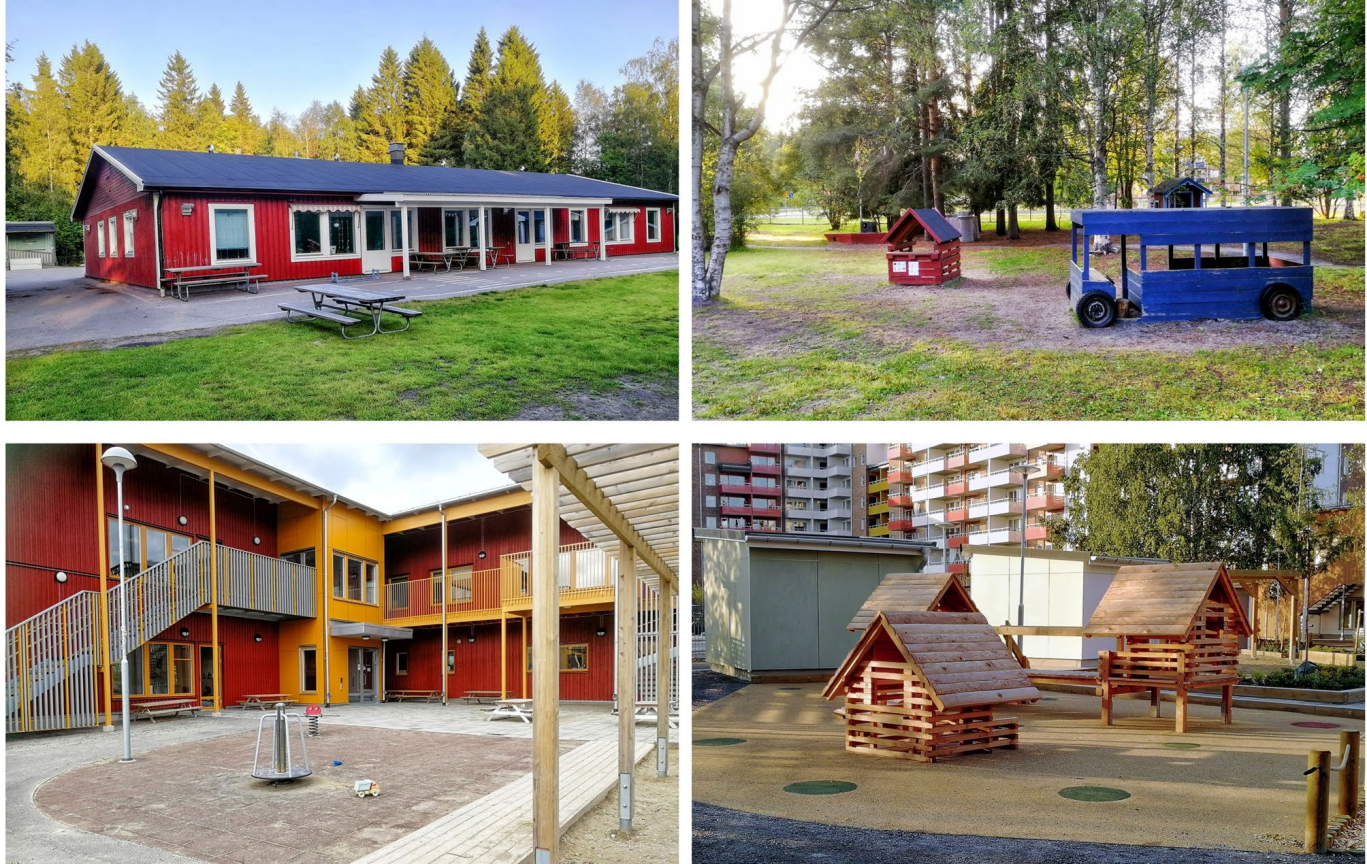
Photos from a typical preschool with a more traditional design, often older preschools (top, left and right) and a typical preschool with a more modern design, often newer preschools (bottom, left and right) included as part of the research project

This cross-sectional study is based on data from the transdisciplinary research project Sustainable encounters in large preschool playgrounds. In total, the research project involved 21 preschools within a municipality in northern Sweden, representing almost half of all available preschools in the municipality. In the overall research project, the 21 preschools were divided into two broad categories relating to the overall design of the buildings and playgrounds:

1. The first category involved preschools with a more traditional design, often older preschools (OPs). These preschools were typically built in one floor. They were characterised by large playgrounds including more green areas often with preserved parts of forest-like nature. The total amount of children in each of these preschools were around 70 divided into 3–5 units. As an example, Fig. 1 presents photo from a typical OP.
2. The second category involved preschools with a more modern design, often newer preschools (NPs). These preschools were typically built in two floors. They were characterised by smaller playgrounds with fewer and more park-like green areas than the traditional preschools. The total amount of children in each of these preschools were around 100 or more divided into 6–8 units. As an example, Fig. 1 presents photo from a typical NP.

**Fig. 2:**
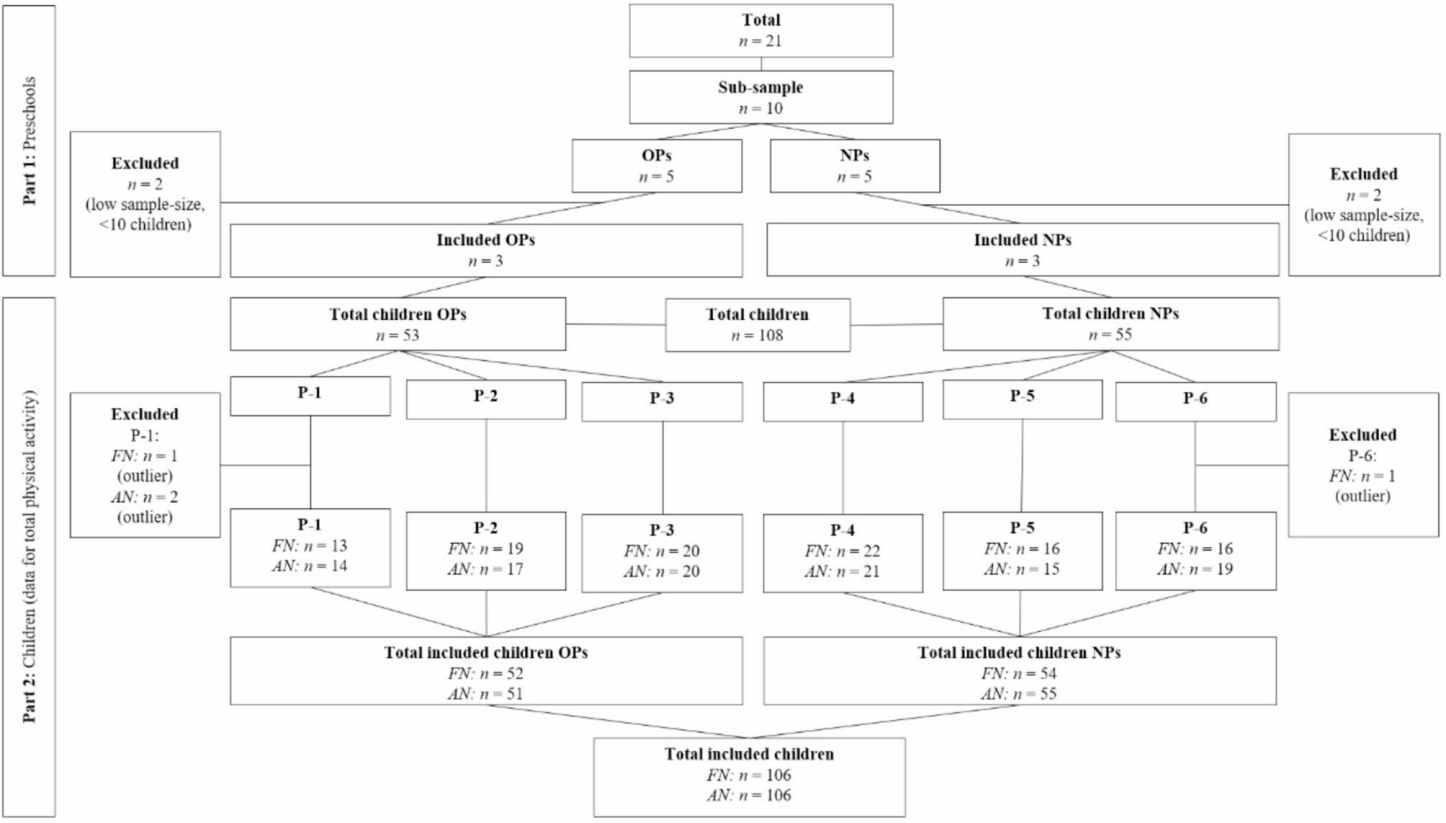
Flow-chart: Part 1 shows the total number of preschools involved in the research project, the selected sub-sample, and the number of preschools excluded and included in the present study. Part 2 displays the total number of children in the selected sub-sample of preschools, along with the number of children included and excluded (Abbreviations: AN, Afternoon; FN, Forenoon; NPs, preschools with a more modern design, often newer preschools; OPs, preschools with a more traditional design, often older preschools)

In total, the research project involved three data collection periods over three years, of which data from the second period was used in the present study. Based on observations and findings from the first data collection period, a sample of ten preschools were selected and included in the second data collection period. Of these ten preschools, five were OPs and five were NPs. The ten preschools were strategically selected as they differed in key characteristics (e.g. total number of children, total outdoor area accessible to the children, and design of the outdoor play environments). As part of the first data collection period (September-October 2021), preschool teachers estimated that the weekly outdoor time was approximately 13–17 h although there were some week-to-week variations depending on different factors and circumstances, such as number of available teachers and present children, as well as the overall organisation (e.g., days or weeks with thematic elements of teaching) and weather conditions. Furthermore, due to small sample sizes with valid data for total PA in four of the ten preschools, data from six preschools– three OPs and three NPs– was included in the present study. Figure 2, part 1 shows the total number of preschools involved in the research project, the selected sub-sample, and the number of preschools excluded and included in the present study.

### Data collection and procedure

For logistical reasons, the data included in this study was collected over three weeks, from September to October 2022. During these weeks, data was collected for total PA using accelerometers, and PA level and type using observation protocols. In addition, descriptive data for preschools and their overall characteristics of outdoor play environment was collected during the first data collection period of the research project.

### Descriptive data for preschools and their overall characteristics of outdoor play environment

Descriptive data for preschools was obtained from the municipal databases. This included data for age groups and total number of children, as well as total accessible outdoor area (m^2^) and accessible outdoor area per child (m^2^). Furthermore, the overall characteristics of outdoor play environment at each preschool were assessed using the Outdoor Play Environment Categories (OPEC) tool that includes three variables scored one, two or three [15]. The total score for each preschool outdoor environment was divided by three, resulting in an average score ranging from one to three. The higher the OPEC score, the higher the quality of the preschool outdoor environments.

The OPEC contains the following three variables:

1. Total outdoor area accessible to the children: a small outdoor area (< 2000 m^2^) is scored as one; a medium (2000–6000 m^2^) as two; and a large (> 6000 m^2^) as three. Information for this variable was obtained from municipal databases.
2. Proportion of the area containing shrubbery, trees or hilly terrain: little or non-existent areas of shrubbery, trees or hilly terrain is scored as one; less than half of the area as two; and more than half of the area as three. Information for this variable was obtained from photos taken during the first data collection period and Google maps.
3. Degree of integration between vegetation, open areas and play structures: no integration (i.e., most vegetation along edges and scanty vegetation adjacent to play structures) is scored as one. Either of the following two criteria is scored as two: (a) play structures adjacent to trees and shrubbery or integrated into areas with the character of wild nature, or (b) the open spaces are located in between play areas and not in separate parts of the environment. Finally, outdoor environments fulfilling both criteria above are scored as three. Similar to above, information for this variable was obtained from photos taken during the first data collection period and Google maps.

Overall, the OPEC tool suggest that large, integrated spaces with abundant greenery and varied topography offer greater play potential than smaller areas where open spaces, vegetation, and play structures are separated [15].

### Total physical activity

Data for outdoor total PA was collected with ActiGraph GT3X+ (ActiGraph LLC, Pensacola, FL, USA) triaxial accelerometers during one week for each preschool. Accelerometers are electronic motion sensors that provide reliable and valid estimates of PA and that have been frequently used in research with children of all ages [16–18].

During each week of data collection, the accelerometers were initiated to collect data from Monday to Friday. The preschool teachers were handed the accelerometers during Monday forenoon and instructed to make sure that the accelerometers were worn by the children on the right hip using an elastic band during all outdoor time, except when they experienced any discomfort. The teachers were also instructed to remove the accelerometers and lay them at the children’s personal site in the dressing room when they went indoors. The researchers collected the accelerometers on Friday afternoon.

The collected accelerometer data was downloaded and processed through ActiLife software (ActiGraph LLC, Pensacola, FL, USA). As the researchers could not simultaneously be present at all preschools, the ActiLife wear-time algorithm was used to estimate outdoor time by removing every ten minutes of consecutive sequences of non-wear-time (representing presumed indoor time as the instructions were to remove the accelerometers when the children went indoors) from the total registered time. The collected data was reviewed and data for Monday forenoon (08:00–12:00 AM) (representing time when the accelerometers were handed out) and Friday afternoon (12:00–17:00 AM) (representing time when the accelerometers were collected) was removed from the analyses. The minimum requirement of accelerometer-data to be included in the analyses was set to one preschool day with ten registered minutes. The measure of PA used in the present study was mean counts per minute (CPM) during outdoor time, representing the total PA during forenoon and afternoon, respectively. CPM was calculated as vector magnitude (the square root of the sum of activity counts squared in each vector) divided by total registered time, with the logic being that the higher the CPM, the higher the total PA.

In total, data from 108 children aged 3–5 years were used for both forenoon and afternoon total PA for OPs (*n* = 53 children) and NPs (*n* = 55 children), respectively. When exploring CPM across the dataset, accelerometer-data from four children was excluded as they were classified as outliers (cutoff: standardized scores > 4.0; observed standardized values: >4.6). Of these, two involved accelerometer-data for forenoon, and two for afternoon. Figure 2, part 2 displays the total number of children in the selected sub-sample of preschools, along with the number of children included and excluded.

### Physical activity level and type

Because hip-worn accelerometers do not reliably detect activities involving minimal hip movement but significant extremity movement, such as those performed while sitting, direct observation was also used to complement the accelerometer data. The PA level and type were assessed using a modified version of the Observational System for Recording Physical Activity in Children– Preschool version (OSRAC-P) [19]. More specifically, PA level (the behavioural form of PAs at different levels) was observed and recorded using five codes: stationary or motionless, stationary with movement of limbs or trunk, slow-easy movements, moderate movements, and fast movements. PA type (the typography of PAs) was observed and recorded using a set of 14 codes, including sit, stand, run, climb, jump/skip, pull/push, roll, swing, and throw, among others.

One researcher visited each preschool for one day. During these visits, the researcher recorded PA level and type by pencil-and-paper methods twice daily (one forenoon and one afternoon), each occasion lasting about 45 min. First, the researchers found themselves a position with a clear vantage point of the PAs taking place in the schoolyard. They then clockwise scanned the schoolyard and recorded PA level and type performed by multiple individual or small groups of children. Each recording was conducted in short sequences (15 s) where the researchers observed and recorded PA level and type before continuing scanning the schoolyard. To ensure that the whole schoolyard was covered, the researchers also changed position during the observations. Prior to data collection, the two researchers involved in data collection aimed to minimize subjectivity and inter-rater variability by jointly observing an outdoor activity session and synchronizing their assessments.

### Statistics

Multiple regression was used to address RQ-1, examining how preschool type (OPs/NPs) and OPEC predicted total PA (CPM), while controlling for child age. Age was included as a continuous variable based on the midpoint of each age group (e.g., children aged 3–4 years were assigned a value of 3.5), since older children typically show higher total PA [20]. The variables OPEC, total accessible outdoor area (m^2^) and accessible outdoor area per child (m^2^) were highly correlated (Pearson’s *r* ≥ 0.86), resulting in multicollinearity issues if included in analyses. The variable OPEC was therefore prioritized in the analyses since it is a complex and valuable outcome when designing preschool outdoor environments. As a result, the regression model’s independent variables were the type of preschool (OP/NP), OPEC, and the children’s mean age. The type of preschool was an indicator variable, with NP classified as zero and OP as one. One regression model was performed for forenoon total PA and one model for afternoon total PA. The separate analyses were performed since children’s total PA can vary between forenoon and afternoon due to differences in daily routines, energy levels, and environmental conditions. Analyzing the periods separately allows for a more accurate assessment of how the outdoor play environment influences activity at different times of day. The regression forced entry method was used for both models. The assumptions for multiple regression were met and investigated using correlations, histograms and Q-Q plots (i.e., linearity, homoscedasticity, independence, and normality for residuals).

Moreover, descriptive data on PA level and type were summarised and presented as relative proportion (%). A chi-square test was used to address RQ-2, exploring differences in PA levels between preschool types (OPs vs. NPs). Data for PA type was not included in the statistical analyses. Instead, the variable provided descriptive insights that supported the interpretation of the findings but did not directly inform the tested hypotheses. All analyses were performed using SPSS (v. 28.0.1.1, IBM) with a 5% alpha level for statistical significance.

### Ethics

The research conducted as part of the research project Sustainable encounters in large preschool playgrounds was reviewed and approved by the Swedish Ethical Review Authority (No. 2021–02403). Prior to data collection, all participants (schools, teachers, children and their parents) received information regarding the aim and procedure of the project. Written informed consent was obtained from the care takers/parents, and they agreed to their children taking part voluntarily. The children were informed in a more practical way, by showing them the accelerometer and instructing them how and why to wear the equipment during their outdoor time. All participants could withdraw their participation at any time without providing any further explanation, for example, if one child did not want to wear the accelerometer one day, s/he was not forced to do so. The collected data was de-identified and handled with strict adherence to ethical guidelines to ensure the protection of individual children.

## Results

### Descriptive data for preschools and the overall characteristics of outdoor play environment

Descriptive data for preschools and their overall characteristics of outdoor play environment are presented in Table 1. The preschools varied in terms of total number of children (range: 72–130), total accessible outdoor area (m^2^) (range: 1208–5729 m^2^), total accessible outdoor area per child (m^2^) (range: 11.5–71.6 m^2^) and OPEC (range: 1.3-3.0). All preschools except one NP exceeded the recommended 3000 m^2^, whereas all OPs and none of the NPs reached the total accessible outdoor area per child of 40 m^2^. Furthermore, among OPs, the total number of children ranged from 72 to 80 and the corresponding figures among NPs were 105–130. Accessible outdoor area (m^2^) per child ranged from 44.6 to 71.6 m^2^ among OPs and 11.5–39.7 m^2^ among NPs, whereas OPEC ranged from 2.3 to 3.0 among OPs and 1.3–2.6 among NPs.

**Table 1:** Descriptive data for preschools and their overall characteristics of outdoor play environment, as well as total physical activity (CPM) across the preschools

Preschool	Descriptive data and the overall characteristics of outdoor play environment					Total physical activity			
			Accessible outdoor area (m^2^)			Study participants (*n*)		CPM (mean, SD)	
	Children at the preschool (*n*)	Age (y)	Total	Per child	OPEC	Forenoon	Afternoon	Forenoon	Afternoon
*OPs*									
P-1	80	4–5	5729	71.6	3.0	13	12	2760 (717)	2447 (783)
P-2	72	3–4	3209	44.6	2.6	19	17	1995 (390)	2012 (472)
P-3	72	4–5	3949	54.8	2.3	20	20	2072 (416)	2571 (549)
*NPs*									
P-4	105	5	1208	11.5	1.3	22	21	2148 (316)	2077 (287)
P-5	130	3–5	4751	36.5	2.6	16	15	2316 (677)	2560 (655)
P-6	108	5	4284	39.7	2.6	16	19	2911 (616)	3038 (588)

Abbreviations: CPM, Counts Per Minute; NP, Preschools with a more modern design, often newer ones; OP, Preschools with a more traditional design, often older ones; OPEC: Outdoor Play Environment Categories

Note: Forenoon: 08:00–12:00 AM; Afternoon: 12:00–17:00 AM

### Total physical activity

The overall mean (SD) number of accelerometer-registered preschool days was 2.7 (1.0) for forenoon with an average of 275 (172) minutes per day, and 2.7 days (1.1) for afternoon with an average of 235 (143) minutes per day.

**Table 2:** The linear model of forenoon and afternoon total physical activity (CPM) predictors, with 95% confidence intervals reported for unstandardised B in parentheses

Time	Variable	B	SE B	β	*p*-value
*Forenoon*	Constant	-1516 (-2955; -78)	725		0.039
	Type of preschool (OP/NP)	-305 (-557; -52)	127	− 0.25	0.019
	OPEC	680 (430; 930)	126	0.61	< 0.001
	Children’s mean age	551 (309; 793)	122	0.50	< 0.001
*Afternoon*	Constant	-1139 (-2737; 459)	806		0.161
	Type of preschool (OP/NP)	-283 (-566; -1)	142	− 0.22	0.049
	OPEC	639 (363; 914)	139	0.53	< 0.001
	Children’s mean age	508 (238; 779)	136	0.42	< 0.001

Abbreviations: β, standardised beta; CPM, Counts Per Minute; NP, Preschools with a more modern design, often newer ones; OP, Preschools with a more traditional design, often older ones; OPEC: Outdoor Play Environment Categories; SE, standard error

#### Forenoon

Descriptive data for forenoon total PA (presented as CPM) is shown in Table 1. As shown in Table 2, the regression model significantly predicted forenoon CPM (F[3, 102] = 12.91, *p* < 0.001). The model explained 28% of the variance in CPM, with an adjusted R^2^ of 0.25. All three independent variables were significant predictors for CPM, where the type of preschool had a negative effect (t[196] = -2.39, *p* = 0.019), while OPEC and children’s age had a positive effect (t[196] = 5.40 and 4.52, respectively, both *p* < 0.001). The regression equation was Y = -1516–305 × 1 + 680 × 2 + 551 × 3, where an OP (X1) decreased CPM with 305 units, an increased rating of one in OPEC (X2) increased CPM with 680 units, and a mean children age increase of one year (X3) increased CPM with 551 units (Table 2).

#### Afternoon

Descriptive data for afternoon total PA (presented as CPM) is shown in Table 1. As shown in Table 2, the same overall results were found for afternoon data as for forenoon. The regression model significantly predicted afternoon CPM (F[3, 102] = 9.29, *p* < 0.001). The model explained 22% of the variance in CPM, with an adjusted R^2^ of 0.19. All three independent variables were significant predictors for CPM, where the type of preschool again had a negative effect (t[196] = -1.99, *p* = 0.049) and OPEC and children’s age had a positive effect (t[196] = 4.60 and 3.72, respectively, both *p* < 0.001). The regression equation was Y = -1139–283 × 1 + 639 × 2 + 508 × 3, where an OP (X1) decreased CPM with 283 units, an increased rating of one in OPEC (X2) increased CPM with 639 units, and a mean children age increase of one year (X3) increased CPM with 508 units (Table 2).

In summary, the results in relation to RQ-1 showed that an OP decreased CPM both during the forenoon (-305 units) and afternoon (-283 units). In addition, an increased rating of one in OPEC increased CPM with 680 units during the forenoon and 639 units during the afternoon.

### Physical activity level and type

Descriptive data for proportion of PA level and type across the six preschools is shown in Table 3. In total, 214 and 157 observations were recorded for PA level in OPs and NPs, respectively. In terms of PA type, 240 observations were recorded for OPs and 168 for NPs.

For the PA level slow-easy movements, the proportions ranged between 45 and 62% for OPs and 44–58% for NPs (Table 3). The proportions of moderate movements ranged between 8 and 38% for OPs and 3–15% for NPs, whereas the corresponding figures for fast movements were 3–10% for OPs and 5–24% for NPs. The chi-square test revealed significant differences between OPs and NPs in PA levels (X2[3, 371] = 12.90, *p* = 0.005), where post-hoc test indicated that OPs had higher proportions of moderate movements compared to NPs (Adjusted residual=-2.95).

In terms of PA types, sit, stand or walk were common across all OPs and NPs (Table 3). The PA type sit ranged between 14 and 20% among OPs and 16–38% among NPs. Stand ranged between 23 and 32% among OPs and 15–29% among NPs, whereas walk ranged between 19 and 32% and 26–34%, respectively. The proportion of the PA type run was relatively low at all OPs and NPs (5–9%) except for one NP (P-6) where it represented 21%. The PA type ride was only observed at one OP (P-2) where it represented 18%. Finally, the proportion of other PA types, including climb/hang, dance, jump/skip, lie down, pull/push, roll, rough and tumble, and swing were 10% or less across both OPs and NPs.

**Table 3:** Proportions of physical activity level and type across the preschools

	OP				NP			
	*P*-1	*P*-2	*P*-3	Total	*P*-4	*P*-5	*P*-6	Total
*Physical activity level*								
**Observations (n)**	**76**	**77**	**61**	**214**	**83**	**41**	**33**	**157**
Stationary or motionless	5%	0%	3%	3%	1%	15%	3%	5%
Stationary with limb or trunk movements	18%	14%	16%	16%	24%	22%	15%	22%
Slow-easy movements	59%	45%	62%	55%	58%	44%	55%	54%
Moderate movements	12%	38%	8%	20%	8%	15%	3%	9%
Fast movements	5%	3%	10%	6%	8%	5%	24%	11%
*Physical activity type*								
**Observations (n)**	**90**	**78**	**72**	**240**	**91**	**39**	**38**	**168**
Climb/hang	7%	4%	4%	5%	3%	8%	10%	6%
Dance	0%	1%	1%	1%	3%	0%	0%	2%
Jump/skip	1%	5%	3%	3%	8%	3%	3%	5%
Lie down	0%	0%	0%	0%	0%	0%	0%	0%
Other*	0%	5%	0%	2%	0%	0%	0%	0%
Pull/push	2%	0%	0%	1%	0%	3%	0%	1%
Ride (e.g., riding a tricycle)	0%	18%	0%	6%	0%	0%	0%	0%
Roll	0%	1%	0%	0%	0%	0%	0%	0%
Rough and tumble	1%	0%	1%	1%	0%	0%	0%	0%
Run	9%	5%	8%	8%	5%	5%	21%	9%
Sit/squat	20%	14%	17%	17%	23%	38%	16%	25%
Stand	29%	23%	32%	28%	29%	15%	16%	23%
Swing	2%	3%	0%	2%	0%	0%	0%	0%
Throw	0%	1%	1%	1%	0%	3%	0%	1%
Walk	29%	19%	32%	27%	29%	26%	34%	29%

Abbreviations: NP, Preschools with a more modern design, often newer ones; OP, Preschools with a more traditional design, often older ones

*Physical activity types other than the options listed

In summary, the results in relation to RQ-2 showed differences in PA levels, where OPs had higher proportions of moderate movements compared to NPs. No differences were observed for the remaining PA levels.

## Discussion

Using a quantitative approach, this study aimed to explore outdoor total PA and PA levels among children in OPs and NPs across different outdoor play environments. The findings were that children in NPs, and children in preschools with higher OPEC, exhibited higher outdoor total PA than their children peers during both the forenoon and afternoon sessions. In addition, that children in OPs had higher proportions of moderate movements compared to NPs.

In the present study, total PA was higher (~ 2000–3000 CPM) than a previous study involving a sample of Swedish preschool children (~ 1000 CPM) but the usage of different accelerometers (uniaxial vs. triaxial) likely explain these findings [12]. The similar explanation may be applied when comparing total PA in the present study to some previous international research For example, Tandon et al. reported lower total PA among children aged 3–5 years from five preschools in USA [21].

The finding that children in NPs had higher outdoor total PA than their children peers in OPs stands in contrast to the RQ-1 hypothesis. Although we are not aware of any studies that directly have compared outdoor total PA at OPs and NPs, some of the characteristics that differed between OPs and NPs was expected to have implications for PA in favour of children at OPs. For example, previous studies shows that larger outdoor playground and lower density of the outdoor play area is associated with more PA [5]. In addition, previous qualitative observations from our research project suggest that NPs may lack diverse equipment and natural environments, whereas the outdoor playground designs and rules to protect immature green areas may restrict children’s engagement with natural elements, thereby possibly having implications for outdoor PA [14]. One possible explanation may be that the nature of specific PAs taking place at the preschools during data collection have implications for the total PA. For one of the NPs (P-6), the high total PA (accelerometers) and notably higher proportion of the PA level fast movements (observation protocols) may be due to soccer play on the yard initiated by a preschool teacher. The yard sloped, resulting in the soccer ball naturally speeding up when rolling downwards, therefore promoting running among the children. In addition, the notably higher proportion of the less intense PA level moderate movements for one OP (P-2) may be due to many children riding tricycles (18% of the observed PA type) both across the yard and in green/forest areas, and this type of activity is known to be less accurately captured by the accelerometer (worn on the hip) and therefore not reflected in the total PA data. Observations also suggested that the size of the preschool (in terms of total number of children) sometimes influenced the organisation of outdoor PAs. For example, as one NP (P-5) hosted a lot of children (*n* = 130), where about 70–80 children were outdoors at the same time, preschool teachers activated the children in different albeit structured ways. In some ways, these teacher-initiated activities may positively be associated with PA yet negatively alter the children’s own development of playfulness and diverse motor abilities. At the same time, however, it should be acknowledged that this also indicates that the teachers were committed and strived to arrange activities that may promote PA and a variation of movement activities among the children.

Moreover, the finding that children at preschools with higher quality of the outdoor play environments had higher total PA than those at preschools with lower quality of the outdoor play environments confirm the RQ-1 hypothesis. Overall, the present study emphasizes the importance of the quality of the preschool outdoor environments, as measured by OPEC, as the strongest predictor for total PA among children, with higher OPEC scores being positively associated with higher total PA for both forenoon and prenoon. The positive association between OPEC scores and PA have been shown in other studies using objective measures of PA [22, 23]. This suggests that environmental features in OPEC, such as integrated green spaces and varied topography, may play a more decisive role in promoting PA than the broad categorization of preschools as OPs or NPs. While differences between OPs and NPs were observed, where children from NPs had higher total PA, these distinctions may, as suggested above, partly reflect variations in specific activities, such as cycling, which accelerometers capture less accurately. Emphasizing OPEC as a predictor of total PA provides a more nuanced understanding of how environmental characteristics influence movement patterns and highlights the methodological challenges in assessing PA across diverse preschool settings. In any case, the similar results for the beta coefficients across the forenoon and afternoon regression analyses strengthen our results of differences in total PA between the two types of preschools and across OPEC ratings. Moreover, the total PA appeared relatively stable between forenoon and afternoon sessions across the six preschools (see Table 1).

In relation to the RQ-2 hypothesis, the findings of the present study were that children in OPs had higher proportions of the outdoor PA level moderate movements compared to NPs, but no differences were found for the remaining PA levels. Overall, the highest proportion of PA level for both OP and NP was stationary with limb or trunk movements or slow-easy movements, covering approximately 60–80% of the observations when combined. Overall, these PA levels correspond with the high proportion of the three PA types sit, stand and walk, and was usually reflected as sitting and playing with spade and bucket in sandboxes, standing while observing other children or events taken place outside the school yard, and walking across play contexts. Furthermore, the discrepancy in results between the two measurement methods (accelerometry and observations) may be partly attributed to differences in data collection periods. While accelerometer data were collected over the course of five consecutive days, the observational assessments were conducted on a single day. As a result, the observational method may be more sensitive to the specific activities that took place during the observation session, which might not be representative of typical behavior. Certain types of PAs, such as cycling, are known to be more difficult to detect accurately with accelerometers. This is particularly relevant when accelerometers are worn on the hip, where they may fail to capture lower-limb movements with sufficient sensitivity.

The present study has both strengths and limitations. In terms of strengths, the data for total PA was collected with accelerometers that have been acknowledged to provide reliable and valid estimates of PA [16–18]. During data collection, however, preschool teachers reported that some children occasionally found the accelerometer to be uncomfortable or intimidating, which may have affected the children’s engagement with the device. At least in part, this may explain the low sample size observed in four of the ten preschools (excluded from the analyses). Furthermore, a strength of this study was the combination of accelerometer and observation protocols that, together with additional fieldwork observations, provided not only objective measures of total PA but also contextual insights that enrich our understanding in relation to the study aim and research question. Although the aim was to minimize subjectivity and inter-rater variability by jointly observing an outdoor activity session and synchronizing their assessments, behaviour mapping and fieldwork observations may be prone to subjectivity and researcher bias, which should be considered when interpreting the findings. As another limitation, the small sample of children wearing accelerometers, as well as the nature of the observation protocols (one-day-visit), limits the possibility for generalization across other OPs and NPs.

Although the findings may not be broadly applicable to other OPs and NPs due to the specificity of the context and participants involved, the present study offer valuable insights that can used for further research. Future studies may explore outdoor PA among children in OPs and NPs, and different types of preschool playgrounds, using a larger sample-size to allow for more robust analyses and conclusions. Furthermore, it would be interesting to explore whether targeted planning of outdoor activities may influence children’s PA, including teachers’ behaviour. In addition, further studies should explore how social dynamics, spatial organization, and activity flow within newly designed preschools across different outdoor play environments contribute to higher total PA among children.

## Conclusions

In conclusion, the present study showed that children in NPs, and children in preschools with higher-quality outdoor play environment, had higher outdoor total PA than their children peers. In addition, that children in OPs had higher proportions of moderate movements compared to NPs. These results emphasise the importance of balancing environmental design and unstructured PAs to support diverse and engaging outdoor PA opportunities for children.

## Data Availability

The datasets used and/or analysed during the current study are available from the corresponding author on reasonable request.

## Abbreviations

CPM: Counts Per Minutes
NP: Newer preschools
OP: Older preschools
OPEC: Outdoor Play Environment Categories
OSRAC-P: Observational System for Recording Physical Activity in Children– Preschool version
PA: Physical Activity

## References

[1] Carson V, Lee EY, Hewitt L, Jennings C, Hunter S, Kuzik N, et al. Systematic review of the relationships between physical activity and health indicators in the early years (0–4 years). BMC Public Health. 2017;17(Suppl 5):854. 29219090 10.1186/s12889-017-4860-0 PMC5753397

[2] Chawla L. Benefits of nature contact for children. J Plan Lit. 2015;30(4):433–52.

[3] Gray C, Gibbons R, Larouche R, Sandseter EB, Bienenstock A, Brussoni M, et al. What is the relationship between outdoor time and physical activity, sedentary behaviour, and physical fitness in children? A systematic review. Int J Environ Res Public Health. 2015;12(6):6455–74. 26062039 10.3390/ijerph120606455 PMC4483711

[4] Veldman SLC, Chin A, Paw MJM, Altenburg TM. Physical activity and prospective associations with indicators of health and development in children aged < 5 years: A systematic review. Int J Behav Nutr Phys Act. 2021;18(1):6. 33413484 10.1186/s12966-020-01072-w PMC7791660

[5] Martin A, Brophy R, Clarke J, Hall CJS, Jago R, Kipping R, et al. Environmental and practice factors associated with children’s device-measured physical activity and sedentary time in early childhood education and care centres: A systematic review. Int J Behav Nutr Phys Act. 2022;19(1):84. 35836231 10.1186/s12966-022-01303-2 PMC9284804

[6] Terrón-Pérez M, Molina-García J, Martínez-Bello VE, Queralt A. Relationship between the physical environment and physical activity levels in preschool children: A systematic review. Curr Environ Health Rep. 2021;8(2):177–95. 33934294 10.1007/s40572-021-00318-4

[7] Sandseter EBH, Rune S, Sando OJ. The dynamic relationship between outdoor environments and children’s play. Educ 3–13. 2022;50(1):97–110.

[8] Swedish National Agency for Education. Curriculum for the preschool (Lpfö 18). 2018. [Internet]. Stockholm: Swedish National Agency for Education. Available from: https://www.skolverket.se/publikationsserier/styrdokument/2018/curriculum-for-the-preschool-lpfo-18

[9] Åström F, Björck-Åkesson E, Sjöman M, Granlund M. Everyday environments and activities of children and teachers in Swedish preschools. Early Child Dev Care. 2022;192(2):187–202.

[10] Sollerhed A-C, Olesen LG, Froberg K, Soini A, Sääkslahti A, Kristjánsdóttir G et al. Movement and physical activity in early childhood education and care policies of five nordic countries. Int J Environ Res Public Health. 2021; 18(24). 10.3390/ijerph182413226 PMC8706902 34948837

[11] Chen C, Ahlqvist VH, Henriksson P, Magnusson C, Berglind D. Preschool environment and preschool teacher’s physical activity and their association with children’s activity levels at preschool. PLoS ONE. 2020;15(10):e0239838. 33057340 10.1371/journal.pone.0239838 PMC7561096

[12] Raustorp A, Pagels P, Boldemann C, Cosco N, Söderström M, Mårtensson F. Accelerometer measured level of physical activity indoors and outdoors during preschool time in Sweden and the united States. J Phys Act Health. 2012;9(6):801–8. 21952100 10.1123/jpah.9.6.801

[13] Boverket. Boverkets allmänna råd (2015:1) om friyta för lek och utevistelse vid fritidshem, förskolor, skolor eller liknande verksamhet. BFS 2015:1; 2025. [Internet]. Available from: https://forfattningssamling.boverket.se/detaljer/BFS2015-1

[14] Manni A, Annerbäck J, Löfgren H, Mårtensson F, Fröberg A. Places, spaces and encounters with nature– socio-material discourses in Swedish preschools. Int J Early Years Educ. 2024:1–19.

[15] Mårtensson F, Boldemann C, Söderström M, Blennow M, Englund JE, Grahn P. Outdoor environmental assessment of attention promoting settings for preschool children. Health Place. 2009;15(4):1149–57. 19643655 10.1016/j.healthplace.2009.07.002

[16] Bruijns BA, Truelove S, Johnson AM, Gilliland J, Tucker P. Infants’ and toddlers’ physical activity and sedentary time as measured by accelerometry: A systematic review and meta-analysis. Int J Behav Nutr Phys Act. 2020;17(1):14. 32028975 10.1186/s12966-020-0912-4 PMC7006115

[17] Hnatiuk JA, Salmon J, Hinkley T, Okely AD, Trost S. A review of preschool children’s physical activity and sedentary time using objective measures. Am J Prev Med. 2014;47(4):487–97. 25084681 10.1016/j.amepre.2014.05.042

[18] Migueles JH, Cadenas-Sanchez C, Ekelund U, Delisle Nystrom C, Mora-Gonzalez J, Lof M, et al. Accelerometer data collection and processing criteria to assess physical activity and other outcomes: A systematic review and practical considerations. Sport Med. 2017;47(9):1821–45. 10.1007/s40279-017-0716-0 PMC6231536 28303543

[19] Brown WH, Pfeiffer KA, McLver KL, Dowda M, Almeida MJ, Pate RR. Assessing preschool children’s physical activity: the observational system for recording physical activity in children-preschool version. Res Q Exerc Sport. 2006;77(2):167–76. 16898273 10.1080/02701367.2006.10599351

[20] Cooper AR, Goodman A, Page AS, Sherar LB, Esliger DW, van Sluijs EM, et al. Objectively measured physical activity and sedentary time in youth: the international children’s accelerometry database (ICAD). Int J Behav Nutr Phys Act. 2015;12:113. 26377803 10.1186/s12966-015-0274-5 PMC4574095

[21] Tandon PS, Saelens BE, Zhou C, Christakis DA. A comparison of preschoolers’ physical activity indoors versus outdoors at child care. Int J Environ Res Public Health. 2018;15(11). 10.3390/ijerph15112463 PMC6265760 30400603

[22] Boldemann C, Blennow M, Dal H, Mårtensson F, Raustorp A, Yuen K, et al. Impact of preschool environment upon children’s physical activity and sun exposure. Prev Med. 2006;42(4):301–8. 16448688 10.1016/j.ypmed.2005.12.006

[23] Boldemann C, Dal H, Mårtensson F, Cosco N, Moore R, Bieber B, et al. Preschool outdoor play environment May combine promotion of children’s physical activity and sun protection. Further evidence from Southern Sweden and North Carolina. Sci Sports. 2011;26(2):72–82.

